# The case of a 72-Year-Old Male with Hematemesis and Pneumomediastinum due to Acute Bacterial Esophageal Necrosis

**DOI:** 10.7759/cureus.5698

**Published:** 2019-09-19

**Authors:** Ethan Karle, Tarang Patel, Armin Krvavac

**Affiliations:** 1 Internal Medicine, University of Missouri Healthcare, Columbia, USA; 2 Pulmonary & Critical Care, University of Missouri Healthcare, Columbia, USA

**Keywords:** pneumomediastinum, acute esophageal necrosis, klebsiella pneumoniae, critical care, esophagogastroduodenoscopy, esophagitis

## Abstract

Acute esophageal necrosis (AEN) is a relatively uncommon presentation of esophagitis. AEN is characterized by black necrotic esophageal tissue and is associated with high mortality rates. We discuss the case of a 72-year-old Caucasian male who was admitted to the medical intensive-care unit (MICU) for evaluation of pneumomediastinum. CT of the chest revealed a right lower lobe consolidation, pneumomediastinum, and marked thickening of the distal esophagus. Vital signs on arrival revealed a temperature of 38.3° Celsius, heart rate of 92 beats per minute, respiratory rate of 30 breaths per minute, blood pressure of 144/65, and oxygen saturation of 97% on 15 liters of supplemental oxygen via non-rebreather. Laboratory studies on arrival were remarkable for a white blood cell (WBC) count of 19.75 x10^9^/L, procalcitonin of 3.53 ng/mL, and C-reactive protein (CRP) level 43.95 mg/dL. The patient was intubated for acute hypoxemic respiratory failure and started on intravenous (IV) pantoprazole as well as broad-spectrum antibiotics for possible pneumonia. Bedside bronchoscopy showed no obvious airway deformities or perforations on inspection but did reveal thick copious secretions that were sent for culture. Thoracic surgery was consulted, and an esophagogastroduodenoscopy (EGD) was performed, which demonstrated no obvious tear or perforation. However, it did show swollen and black mucosa primarily involving the distal esophagus. Tissue cultures from the EGD grew Klebsiella pneumoniae, which was also grown from the bronchial wash and bronchoalveolar lavage. EGD findings were consistent with AEN. Despite extensive supportive care, the patient ultimately expired. We propose that people with AEN who present with pneumomediastinum and those in whom AEN is found to be secondary to a bacterial cause require not only supportive measures but also prompt surgical consultation.

## Introduction

Esophagitis is a relatively common diagnosis that can be life-threatening in its most severe form. Acute esophageal necrosis (AEN) is characterized by black necrotic esophageal tissue that is noted on esophagogastroduodenoscopy EGD [1]. The mechanism of injury for AEN remains elusive. The prevailing theories ascribe it to local ischemia, compromised mucosal defenses, and corrosive reflux injury of gastric contents [2]. However, AEN excludes cases secondary to caustic ingestion [3]. AEN is a rare form of esophagitis with prevalence rates between 0.0125% to 0.2%. It typically affects Caucasian males over the age of 60 years with comorbidities such as vascular diseases, diabetes mellitus, alcoholism, and poor nutritional status (albumin level of <3g/dL) [4].

## Case presentation

**Figure 1:**
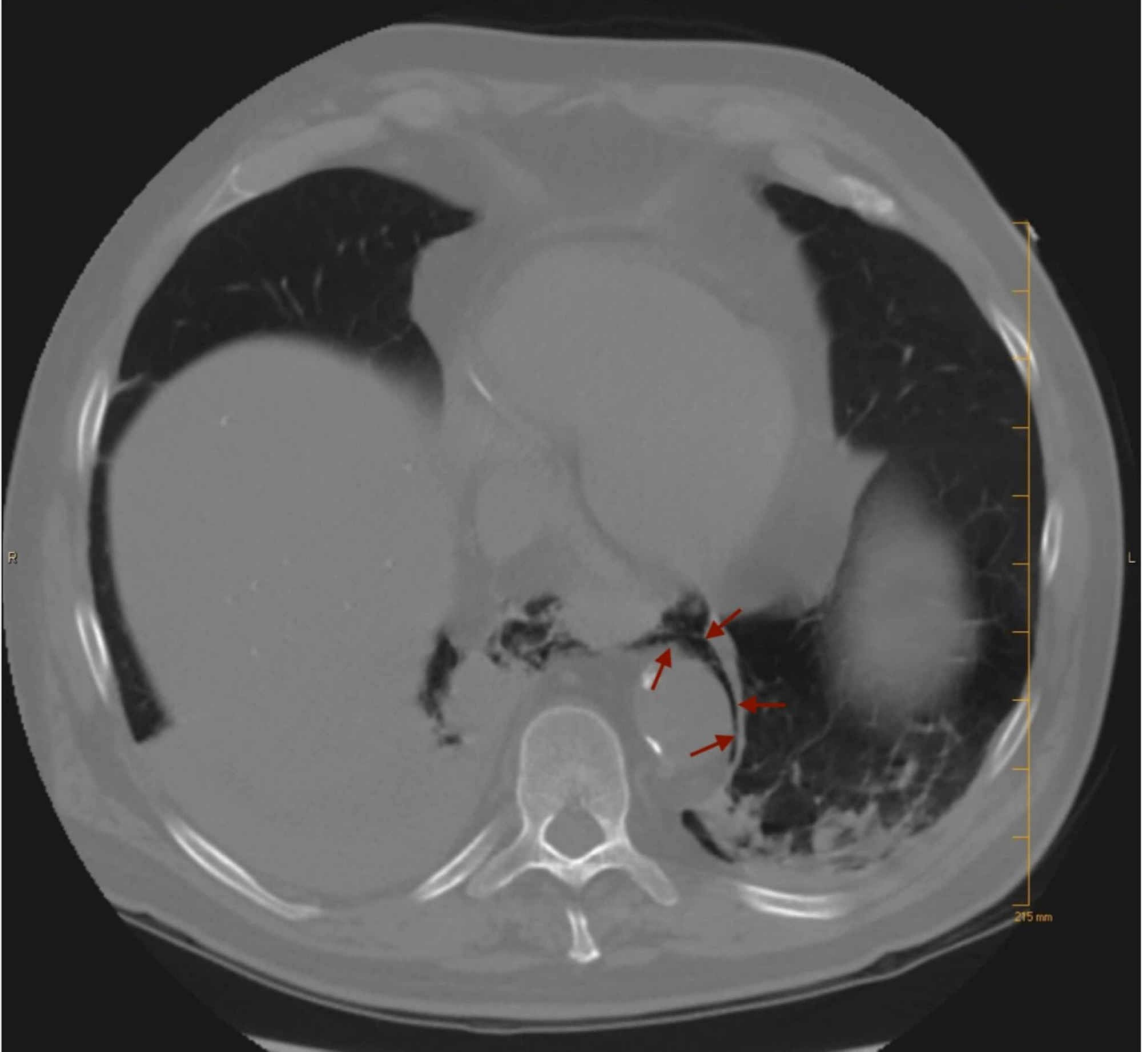
CT of the chest showing pneumomediastinum

**Figure 2:**
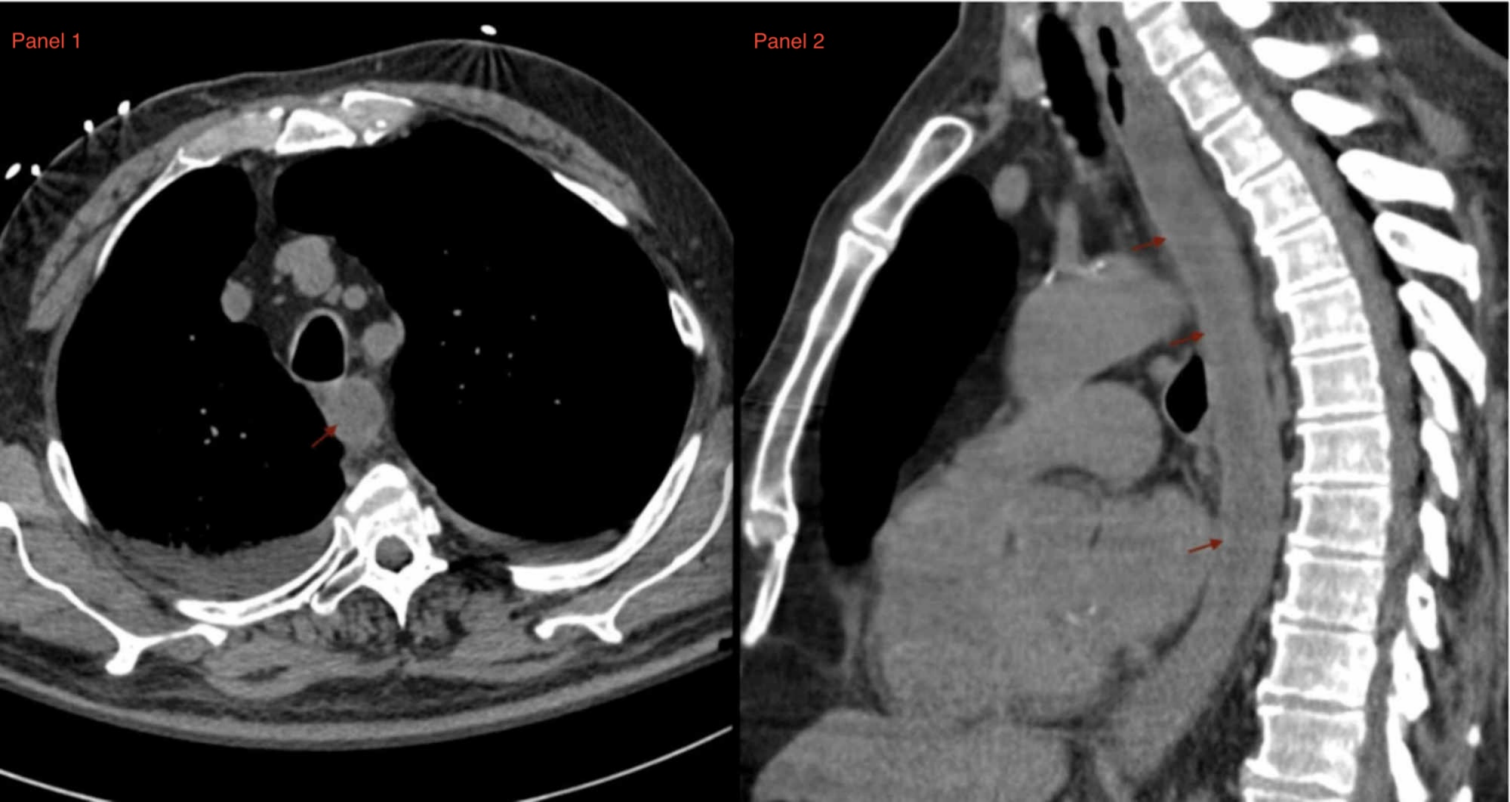
Panel 1: axial CT of the chest showing distal esophageal thickening. Panel 2: sagittal CT of the chest demonstrating distal esophageal thickening

The patient was a 72-year-old Caucasian male with a past medical history of long-standing gastroesophageal reflux disease, tobacco dependence (over 50 packs/year), cerebrovascular disease, and chronic kidney disease. He was admitted to the medical intensive-care unit (MICU) for the evaluation of pneumomediastinum. Two weeks before admission, the patient had been seen in a community emergency department with a three-day history of fevers, dyspnea, and cough. He had been diagnosed with aspiration pneumonia and treated with amoxicillin-clavulanic acid. The patient had subsequently returned to the emergency department two days later with worsening symptoms and hematemesis. At the MICU, a CT of the chest showed a right lower lobe consolidation and pneumomediastinum (Figure 1). The pneumomediastinum tracked from the right paratracheal region along the right lateral aspect of the esophagus to the gastroesophageal junction. There was also a marked thickening of the distal esophagus (Figure 2). Vital signs on arrival included a temperature of 38.3° Celsius, a heart rate of 92 beats per minute, respiratory rate of 30 breaths per minute, blood pressure of 144/65, and an oxygen saturation of 97% on 15 liters of supplemental oxygen via non-rebreather. Physical examination revealed bilateral diffuse rhonchi. Laboratory studies on arrival were remarkable for a white blood cell (WBC) count of 19.75 x109/L, hemoglobin of 13.8 g/dL, and platelet count of 354 x109/L. Renal function was diminished with a serum creatinine of 1.69 mg/dL (baseline: 1.4 mg/dL) and blood urea nitrogen of 39 mg/dL. Abnormal serum chemistry lab results included albumin level of 2.9 g/dL, and liver enzymes were elevated with an alkaline phosphatase of 172 units/L, aspartate aminotransferase and alanine aminotransferase at 112 units/L and 66 units/L, respectively. Procalcitonin and C-reactive protein (CRP) were both elevated at 3.53 ng/mL and 43.95 mg/dL, respectively. Arterial blood gas on a 60% fraction of inspired oxygen (FiO2) demonstrated a pH of 7.472, pCO2 of 32.1 mmHg, pO2 of 72.7 mmHg, and HCO3 of 23.2 mmol/L. The alveolar-arterial gradient was elevated at 315 mmHg. 

The differential diagnosis of pneumomediastinum in the setting of esophageal wall thickening includes Boerhaave’s syndrome (retching- associated esophageal rupture), malignancy-associated esophageal rupture, infectious infiltration of the esophagus, and esophagitis-related esophageal rupture. Initial management of our patient included intubation for increased respiratory rate and acute hypoxemic respiratory failure. Once intubated, the patient was placed on a strict nil-per-os (NPO) status. Intravenous (IV) pantoprazole was scheduled every 12 hours, and empiric broad-spectrum antibiotic therapy was started for suspected pneumonia after bedside bronchoscopy. Bronchoscopy showed no obvious airway deformities or perforations on inspection but did reveal thick copious secretions that were therapeutically aspirated and sent for culture.

**Figure 3:**
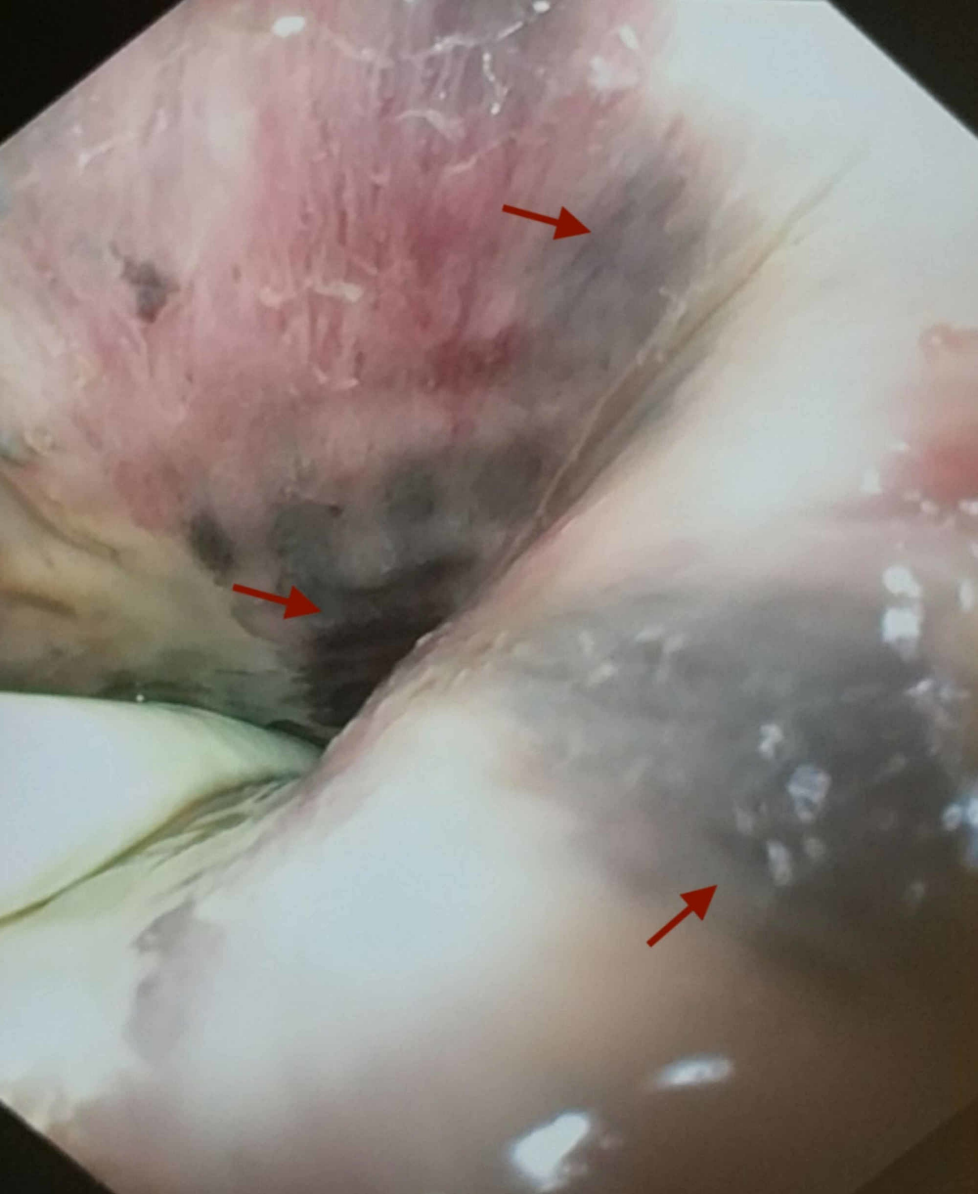
Esophagogastroduodenoscopy (EGD) showing swollen, black mucosa primarily involving the distal esophagus

A Gastrografin (Schering AG, Berlin, Germany) study was ordered but could not be performed as nasogastric or orogastric tubes could not be placed due to concern for esophageal perforation. Thoracic surgery was consulted and an esophagogastroduodenoscopy (EGD) was performed in the operating room. EGD demonstrated no obvious tear or perforation but did show swollen and black mucosa primarily involving the distal esophagus (Figure 3). The gastric mucosa was unaffected. Tissue cultures from the EGD grew Klebsiella pneumoniae, which was also grown from the bronchial wash and bronchoalveolar lavage (Table 1). EGD findings were consistent with acute esophageal necrosis (AEN), and the patient continued to remain NPO. Total parenteral nutrition was considered but deferred as a jejunostomy tube had been placed by thoracic surgery to provide the patient with enteral nutrition to promote healing. Though surgical esophagectomy was considered by the thoracic surgeons, the patient was ultimately deemed to be a poor surgical candidate given his multiple comorbidities. 

**Table 1:** Table comparing culture susceptibility and sensitivity of Klebsiella pneumoniae isolated from right middle lobe bronchoalveolar lavage and tissue biopsy from EGD EGD: Esophagogastroduodenoscopy

Antibiotic susceptibility		
	Culture Source and isolation	
	Right middle lobe bronchoalveolar lavage: Klebsiella pneumoniae	Tissue Biopsy from esophagogastroduodenoscopy: Klebsiella pneumoniae
Antibiotic	Susceptibility	
Amikacin	Sensitive	Sensitive
Ampicillin	Resistant	Resistant
Ampicillin/sulbactam	Sensitive	Sensitive
Cefepime	Sensitive	Sensitive
Cefoxitin	Sensitive	Sensitive
Ceftriaxone	Sensitive	Sensitive
Ciprofloxacin	Sensitive	Sensitive
Gentamicin	Sensitive	Sensitive
Levofloxacin	Sensitive	Sensitive
Meropenem	Sensitive	Sensitive
Piperacillin/tazobactam	Sensitive	Sensitive
Trimethoprim/sulfamethoxazole	Sensitive	Sensitive

The patient’s hospitalization was complicated by critical limb ischemia of the right lower extremity, which prompted the use of a heparin drip. Unfortunately, the patient developed a gastrointestinal bleed while on therapeutic anticoagulation. After a goals-of-care discussion with the patient’s family, the decision was made to extubate the patient to focus on comfort measures. The patient passed away peacefully soon thereafter.

## Discussion

The pathogenesis for the extensive necrosis seen in AEN is yet to be fully understood. Prevailing theories suggest that an ischemic component, compromised mucosal defenses, and corrosive reflux injury of gastric contents are involved [2]. The overall prognosis of AEN is grim as 32% of the patients will succumb to the disease [2]. In those cases where AEN is related to ischemia or corrosive reflux injury, the mainstay of treatment is acid suppression, supportive therapy, and medical management of the underlying process. Of those who survive the underlying process, 10-25% will develop esophageal strictures with the rest experiencing complete resolution of AEN [2].

Once AEN is suspected, supportive interventions should be quickly initiated. Timely fluid resuscitation, acid suppression, and applying a strict NPO status are critical in the early management of uncomplicated AEN. Early initiation of feeding to aid in the healing process should be considered as patients with AEN tend to have a poor nutritional status at baseline [4,5]. Medical management of uncomplicated AEN targets acid suppression either by IV proton-pump inhibitors or histamine-2 receptor blockers. Sucralfate may also be used as monotherapy or as adjunctive therapy with proton-pump inhibitors or histamine-2 receptor blockers [5]. Empiric antimicrobials do not have a role in the treatment of uncomplicated AEN. Antimicrobial therapy should only be initiated if esophageal cultures are positive, if esophageal rupture is suspected, or if there is rapid clinical decline [2,5,6]. In summary, medical management of AEN includes prompt hemodynamic resuscitation, applying strict NPO status, early nutrition, and acid suppression.

Surgical intervention is not usually recommended for uncomplicated AEN. However, surgical consultation for esophagectomy should be obtained for those who develop perforation, mediastinitis, or pneumomediastinum [2,3,6]. Additionally, surgical intervention may play a role in the treatment for a subset of AEN resulting from an infection. There are very few reported cases of Klebsiella pneumoniae being implicated as the cause of AEN [7,8]. In a cohort of patients with AEN secondary to an infectious cause, the overall mortality reached 48%. Furthermore, those treated with antibiotics alone (without surgical intervention) had a mortality rate as high as 90%, while those who underwent surgical intervention had a mortality of only 27% [7]. It is important that the diagnosis of AEN be made in a timely manner in order to promptly initiate appropriate treatment.

## Conclusions

In older Caucasian males with a history of vascular disease, hematemesis, and poor nutrition, AEN should remain on the differential spectrum as it lends itself to worse outcomes and the underlying pathology may require differing treatments. Empiric antibiotic therapy and prompt surgical evaluation for esophagectomy should be considered, particularly in patients with AEN who present with pneumomediastinum and are found to have AEN secondary to a bacterial infection.
